# Thyroid FT4-to-TSH ratio in the first trimester is associated with gestational diabetes mellitus in women carrying male fetus: a prospective bi-center cohort study

**DOI:** 10.3389/fendo.2024.1427925

**Published:** 2024-11-29

**Authors:** Shuoning Song, Yuemei Zhang, Xiaolin Qiao, Yanbei Duo, Jiyu Xu, Jing Zhang, Yan Chen, Xiaorui Nie, Qiujin Sun, Xianchun Yang, Ailing Wang, Zechun Lu, Wei Sun, Yong Fu, Yingyue Dong, Tao Yuan, Weigang Zhao

**Affiliations:** ^1^ Department of Endocrinology, Key Laboratory of Endocrinology of Ministry of Health, Peking Union Medical College Hospital, Chinese Academy of Medical Science and Peking Union Medical College, Beijing, China; ^2^ Department of Obstetrics, Haidian District Maternal and Child Health Care Hospital, Beijing, China; ^3^ Department of Obstetrics, Beijing Chaoyang District Maternal and Child Health Care Hospital, Beijing, China; ^4^ Core Facility of Instrument, Institute of Basic Medical Sciences, Chinese Academy of Medical Sciences, School of Basic Medicine, Peking Union Medical College, Beijing, China; ^5^ Department of Laboratory, Haidian District Maternal and Child Health Care Hospital, Beijing, China; ^6^ Department of Clinical Laboratory, Beijing Chaoyang District Maternal and Child Health Care Hospital, Beijing, China; ^7^ National Center for Women and Children’s Health, China Centers for Disease Control and Prevention (CDC), Beijing, China

**Keywords:** gestational diabetes mellitus, thyroid function, glucose and lipids metabolisms, pregnancy, fetal sex

## Abstract

**Background:**

Gestational diabetes mellitus (GDM) is one of the most common medical complications of pregnancy, which increases the risk of other pregnant complications and adverse perinatal outcomes. Thyroid dysfunction is closely with the risk of diabetes mellitus. However, the relationship between euthyroid function in early pregnancy and GDM is still controversial.

**Aims:**

This study was to find the relationship between thyroid function within normal range during early pregnancy as well as glucose and lipids metabolisms as well as the risk of subsequent GDM.

**Methods:**

A total of 1486 pregnant women were included in this prospective double-center cohort study. Free thyroxine (FT4), thyroid stimulating hormone (TSH) and antithyroid peroxidase antibodies (TPOAb) were tested during 6-12 weeks of gestation and oral glucose tolerance test (OGTT) was conducted during 24-28 weeks to screen GDM. Relative risks (RR) with 95% confidence intervals (CI) for subsequent risk of GDM by thyroid function quartiles were assessed adjusting for major risk factors.

**Results:**

The incidence of GDM was 23.0% (342/1486). TSH, FT4 and the percentage of positive TPOAb were no significant difference between women with and without GDM, but FT4/TSH ratio was significantly higher in GDM group compared with NGT group [6.97(0.84,10.61) vs. 4.88(0.66,12.44), *P*=0.025)]. The linear trends of TC, TG, HDL-C, LDL-C, fasting glucose in the first trimester, insulin, C-peptide, HOMA-IR, fasting glucose during OGTT and incidence of GDM according to FT4/TSH ratio were all statistically significant. Further analysis based on fetal sex presented only the third quartile of FT4/TSH ratio in women carrying male fetus was associated with higher incidence of GDM statistically significant [RR (95% CI), 1.917 (1.143,3.216)], rather than in women carrying female fetus.

**Conclusions:**

Thyroid function even in normal range is closely related to glucose and lipids metabolisms during the first trimester. Unappropriated FT4/TSH ratio in the first trimester is an independent risk factor of GDM in women carrying male fetus.

## Introduction

Gestational diabetes mellitus (GDM) is a state restricted to pregnant women whose impaired glucose tolerance is first discovered during pregnancy (1), which is one of the most common medical complications of pregnancy (2). With the development of economics and increasing of elderly pregnant women, the prevalence of GDM has been growing worldwide in the past years and the increase of GDM incidence in China is extremely alarming (3). GDM increases the risk of pregnancy complications and adverse perinatal outcomes, such as pregnancy-induced hypertension (PIH), premature delivery, macrosomia and so on. It also influences the long-term health of the mother and fetus (4, 5), leading to a higher risk of metabolic disorders in future. Thus, more and more studies have paid attention on finding risk factors of GDM, which may help to predict and prevent GDM.

Thyroid function is closely related to metabolism and thyroid dysfunction during pregnancy can increase the incidence of complication of pregnancy and adverse perinatal outcomes as well as affect infant thyroid function and growth (6). Recently some researchers have found different levels of thyroid function, even within normal range, might affect the maternal metabolism and infant growth (7). However, the relationship between different thyroid indicators and GDM is still evidentiary uncertainty and controversial. Thus, in this study, we aimed to assess the association between euthyroid function and glucose as well as lipids metabolisms during the early pregnancy and explore the risk factors of GDM. Since previous studies found the relationship between maternal thyroid function and fetal growth might be modified by fetal sex (7), sex-specific effect was also taken into consideration in this study.

## Materials and methods

This study was a part of the prospective bi-center cohort study, which aims to find the biological markers of GDM in early pregnancy based on urinary proteomics and establish an effective model to predict GDM as earlier as possible.

At baseline, participants underwent a clinical investigation at the first prenatal visit in early pregnancy (6-12 weeks’ gestation). This prospective study started on 2019 in Haidian District Maternal and Child Health Care Hospital and Chaoyang District Maternal and Child Health Care Hospital, Beijing, China. For all participants in the present study, all available clinical and laboratory data were recorded and verified by two researchers at the same time.

### Study participants

Inclusion and exclusion criteria of participants were as follows. Inclusion criteria (1): gestation age at entry <12 weeks; (2) without diabetes mellitus before pregnancy; (3) acceptance of participation in the study, and signature of the consent form. Exclusion criteria: (1) gemellary or multiple pregnancy; (2) fasting blood glucose≥5.1mmol/L at baseline; (3) any acute or other chronic disease, such as severe liver and renal dysfunction, heart disease, autoimmune disease, and so on. On this basis, a total of 1486 pregnant women with clinical and laboratory data at 6-12 weeks’ gestation and 75g oral glucose tolerance test (OGTT) at 24-28 weeks’ gestation were included in the present study. Perinatal database of 1237 women was collected from electrical medical records (EMR).

The ethics committees of all participating centers approved the study protocol. The study was conducted under the guidance of Major New Drugs Innovation and Development Program (clinical trial number is NCT03246295). Written informed consent was obtained from each participant and the study was performed in accordance with the Declaration of Helsinki as revised in 2013.

### Measurements

Participants were measured body weight and height at the first prenatal visit (6-12 weeks’ gestation) and body weight was monitored during the whole course. Body mass index (BMI) was determined by dividing body weight in kilograms by height in meters squared. Measurements of systolic blood pressure (SBP) and diastolic blood pressure (DBP) were taken by trained nurses with an automatic blood pressure monitor. Medical history, personal history and family history were asked by attending doctors and recorded in EMR. Blood samples were collected at the first prenatal visit to examine liver function, renal function and thyroid function. 75g OGTT was conducted for all participants during 24-28 weeks’ gestation and GDM was diagnosed according by the International Association of the Diabetes and Pregnancy Study Groups criterion in 2010 (8). Perinatal and neonatal outcomes were: pregnancy-induced hypertension (PIH) (including preeclampsia or eclampsia), premature delivery (<37 weeks), caesarean section, postpartum hemorrhage (PPH) (blood loss from the genital tract of ≥500 mL after giving birth) (9), LGA (large for gestational age) and SGA (small for gestational age).

### Statistical analysis

Analyses were conducted using the statistical program SPSS (version 24, SPSS, Chicago, IL). Continuous variables were tested for normality of distribution. Variables with approximately normal distributions were presented as mean ± SD, and those with skewed distributions were presented as median and interquartile range (25th–75th percentile). Categorical variables were presented as percentage (number). Two-sample Student *t* test or Mann-Whitney test was used for continuous variables and χ2 test was used for categorical variables. To determine whether there was a significant graded increase in glucose and lipids level as well as the risk of GDM with increasing FT4/TSH ratio quartiles, the *P* value for the linear trend was calculated. The association of FT4/TSH ratio and GDM were examined by binary logistic regression analysis. To assess confounding factors, covariates including age, preBMI, lipids levels and family history of diabetes were entered into the logistic regression model. Propensity score matching (PSM) analysis was conducted using the R language. Sensitivity analysis was used to evaluated the effect of the follow-up. Statistical significance was inferred from two-sided *P* values <0.05.

## Results

Among 1486 pregnant women, the incidence of GDM was detected as 23.0% (342/1486) in this cohort. 1237(83.2%) pregnant women with perinatal outcomes were recorded in the cohort. The baseline characteristics, perinatal outcomes and neonatal characteristics of the pregnant women with GDM and normal glucose tolerance (NGT) were presented in **Table 1**. Age, BMI during the whole pregnancy, alanine aminotransferase (ALT), total cholesterol (TC), triglyceride (TG), low density lipoprotein cholesterol (LDL-C), glucose in 1^st^ trimester, insulin, C-peptide, fasting blood glucose (FBG) level during OGTT, FT4/TSH ratio, percentage of premature delivery and caesarean delivery were all significantly higher in GDM group than in NGT group. The percentage of positive TPOAb were similar in two groups. We also found FT4/TSH ratio in women carrying female fetus was significantly higher than in male group [(5.84(0.73,13.31) vs. 5.12(0.63,11.52), *P*=0.023)]. TSH was lower in female group, but there was no significant difference [1.60(0.91,2.42) vs. 1.69(1.09,2.48), *P*=0.051]. The percentage of GDM was similar in women carrying female and male fetus (23.2% vs. 22.9%, *P=*0.902). The detailed data were shown in **Supplementary Table 1**.

**Table 1:** Baseline characteristics, perinatal outcomes and neonatal characteristics between women with and without GDM.

Variables	Total	GDM	NGT	*P* value
**Baseline characteristics**	n=1486	n=342	n=1144	
Age(year)	30.39 ± 3.97	31.49 ± 3.97	30.06 ± 3.91	<0.001
BMI in 1^st^ trimester(kg/m^2^)	22.02 ± 3.06	23.06 ± 3.41	21.70 ± 2.88	<0.001
BMI in 2^nd^ trimester(kg/m^2^)	24.51 ± 3.11	25.50 ± 3.42	24.22 ± 2.95	<0.001
BMI in 3^rd^ trimester(kg/m^2^)	26.89 ± 3.12	27.31 ± 3.38	26.76 ± 3.02	0.011
Family history of diabetes mellitus	14%(208)	17.5%(60)	12.9%(148)	0.031
Previous adverse pregnancy outcomes	18.9%(281)	21.9%(75)	18.0%(206)	0.104
SBP(mmHg)	116.87 ± 8.73	116.97 ± 8.64	116.83 ± 8.76	0.830
DBP(mmHg)	74.40 ± 6.70	74.67 ± 6.97	74.32 ± 6.62	0.440
ALT(U/L)	13.0(10.0,18.0)	15.0(11.0,20.86)	12.40(9.9,17.0)	<0.001
AST(U/L)	16.0(14.0,19.0)	16.24(14.4,19.2)	16.0(14.0,18.9)	0.198
TBil(umol/L)	10.6(8.6,13.2)	10.4(8.5,13.2)	10.7(8.7,13.2)	0.493
TC(mmol/L)	3.91(3.44,4.47)	4.01(3.49,4.61)	3.89(3.43,4.43)	<0.001
TG(mmol/L)	0.84(0.64,1.16)	0.95(0.73,1.29)	0.82(0.62,1.11)	0.004
HDL-C(mmol/L)	1.44(1.24,1.64)	1.40(1.19,1.60)	1.45(1.24,1.66)	<0.001
LDL-C(mmol/L)	2.01(1.67,2.43)	2.10(1.73,2.61)	1.97(1.65,2.39)	<0.001
Glucose(mmo/L)	4.50(4.10.4.90)	4.80(4.40,5.20)	4.40(4.10,4.80)	<0.001
Iinsulin(uU/mL)	6.40(4.40,9.10)	8.00(5.10,11.2)	6.10(4.30,8.40)	<0.001
C-Peptide(ng/dL)	0.84(0.63,1.14)	0.98(0.73,1.32)	0.81(0.61,1.07)	<0.001
HOMA-IR	1.29(0.87,1.88)	1.70(1.05,2.43)	1.22(0.84,1.72)	<0.001
OGTT				
FBG(mmo/L)	4.86(4.43,4.91)	5.17(4.84,5.34)	4.59(4.39,4.78)	<0.001
1-h BG(mmo/L)	7.60(6.52,8.69)	9.75(8.34,10.61)	7.30(6.29,5.61,6.96)	<0.001
2-h BG(mmo/L)	6.52(5.77,7.36)	8.03(7.00,8.99)	6.26(5.61,6.96)	<0.001
GDM	23.0%(342)	–	–	
FT4(ng/dL)	1.24(1.12,1.39)	1.24(1.12,1.40)	1.24(1.12,1.39)	0.762
TSH(uIU/mL)	1.63(1.00,2.40)	1.69(1.05,2.40)	1.61(0.99,2.40)	0.322
FT4/TSH ratio	5.54(0.69,12.68)	6.97(0.84,10.61)	4.88(0.66,12.44)	0.025
TPOAb(IU/mL)	11.3%(152/1340)	11.1%(33/298)	11.4%(119/1042)	0.868
**Perinatal outcomes**	n=1237	n=285	n=952	
PIH	7.8%(97)	9.1%(26)	7.5%(71)	0.359
Premature delivery	3.8%(47)	6.3%(18)	3.0%(29)	0.011
Caesarean delivery	32.6%(403)	43.2%(123)	29.4%(280)	<0.001
PPH	13.9%(172)	11.2%(32)	14.7%(140)	0.137
Neonatal outcomes				
Birth age(weeks)	39.29(38.57,40.14)	39.00(38.29,39.86)	39.57(38.71,40.14)	<0.001
Birth weight (g)	3300(3020,3560)	3330(3020,3570)	3300(3020,3545)	0.566
LGA	12.5%(155)	17.5%(50)	11.0%(105)	0.004
SGA	7.8%(96)	14.4%(41)	5.8%(55)	<0.001

P<0.05 represents significant difference between two groups.

Reference range: FT4 0.81-1.69 ng/dL;TSH 0.09-3.99 uIU/ml.

GDM, gestational diabetes mellitus; NGT, normal glucose tolerance; BMI, body mass index; SBP, systolic blood pressure; DBP, diastolic blood pressure; ALT, alanine aminotransferase; AST, aspartate aminotransferase; TBil, total bilirubin; TC, total cholesterol; TG, triglyceride; LDL-C, low density lipoprotein cholesterol; HDL-C, high density lipoprotein cholesterol; HOMA-IR, homeostasis model assessment of insulin resistance; OGTT, oral glucose tolerance rest; FBG, fasting blood glucose; FT4, free thyroxine; TSH, Thyroid Stimulating Hormone; TPOAb, Thyroid peroxidase antibodies; PIH, pregnancy-induced hypertension; PPH, postpartum hemorrhage; LGA, large for gestational age; SGA, small for gestational age.

Baseline characteristics: Baseline characteristics of pregnant women, “n” means the number of participants in each group.

Perinatal outcomes: Perinatal outcomes of pregnant women, “n” means the number of participants in each group.

To examine glucose metabolism, lipids metabolism and the risk of GDM according to FT4/TSH ratio in 1^st^ trimester in further detail, subjects were divided into quartiles of FT4/TSH ratio based on the distribution among the pregnant women (quartile 1, ≤0.69; quartile 2, 0.69-5.54; quartile 3, 5.54-12,68; and quartile 4, ≥12.68). The linear trends of TC, TG, HDL-C, LDL-C, FBG in 1^st^ trimester, insulin, C-peptide, HOMA-IR, fasting glucose during OGTT and incidence of GDM according to FT4/TSH ratio were all statistically significant. The highest level of FBG in 1^st^ trimester, insulin, C-peptide, HOMA-IR, fasting glucose during OGTT and incidence of GDM were in the third quartile of FT4/TSH ratio, while the highest lipids levels were all in the second quartile of FT4/TSH ratio. The detailed results were shown in **Table 2**.

**Table 2:** Glucose and lipids metabolism according to FT4/TSH ratio quartiles.

Variables	Quartile 1	Quartile 2	Quartile 3	Quartile 4	*P* for linear trend
n	369	374	372	371	*-*
FT4/TSH ratio range	≤0.69	0.69-5.54	5.54-12.68	≥12.68	–
OGTT					
FBG(mmo/L)	4.63(4.52,4.84)	4.63(4.40,4.84)	4.75(4.52,5.00)	4.70(4.48,4.95)	<0.001
1-h BG(mmo/L)	7.44(6.51,8.55)	7.48(6.50,8.58)	7.57(6.55,8.72)	7.77(6.59,8.81)	0.432
2-h BG(mmo/L)	7.05(6.13,7.31)	6.52(5.66,7.38)	6.47(5.79,7.26)	6.45(5.77,7.29)	0.541
GDM	19.2%(71)	20.3%(76)	28%(104)	24.5%(91)	0.018
TC(mmol/L)	3.45(3.13,3.99)	4.08(3.63,4.56)	3.80(3.36,4.47)	3.76(3.24,4.31)	<0.001
TG(mmol/L)	0.51(0.38,0.73)	0.86(0.68,1.23)	0.80(0.61,1.07)	0.79(0.60,1.06)	<0.001
HDL-C(mmol/L)	1.32(1.13,1.53)	1.47(1.29,1.69)	1.38(1.18,1.59)	1.36(1.16,1.60)	<0.001
LDL-C(mmol/L)	1.85(1.66,2.13)	2.12(1.77,2.56)	1.98(1.63,2.43)	1.86(1.52,2.26)	<0.001
Glu(mmol/L)	4.55(4.20,5.10)	4.40(4.00,4.80)	4.70(4.30,5.20)	4.70(4.30,5.20)	<0.001
Iinsulin(uU/mL)	4.85(3.95,6.00)	6.20(4.30,8.90)	7.25(4.90,10.30)	6.20(4.30,8.30)	0.010
C-Peptide(ng/dL)	0.74(0.60,0.91)	0.83(0.65,1.14)	0.89(0.63,1.26)	0.78(0.57,1.02)	<0.001
HOMA-IR	1.03(0.74,1.35)	1.23(0.78,1.78)	1.52(1.02,2.19)	1.26(0.90,1.78)	<0.001

The crude and multivariable-adjusted relative risk (RR) of GDM determined by FT4/TSH ratio quartiles were showed in **Table 3**. There was higher RR of GDM in the third and fourth quartile of FT4/TSH ratio after adjustment for age, preBMI, lipids levels and family history of diabetes mellitus (quartile 1, reference; quartile 2, RR 1.219 [95% CI 0.824,1.803]; quartile 3, 1.873 [1.276,2.748]; and quartile 4, 1.608 [1.073,2.409]). However, further analysis showed only the third quartile of FT4/TSH ratio was associated with higher incidence of GDM in women carrying male fetus [quartile 3, 1.917 (1.143,3.216)] rather than in women carry female fetus. 229 pregnant women in GDM group and 421 corresponding pregnant women in NGT group were obtained by propensity score matching (PSM). The baseline characteristics of age, preBMI, the percentage of family history of diabetes mellitus and lipids level were no significant difference between two groups. There was higher RR of GDM in the third and fourth quartile of FT4/TSH ratio only in women carrying male fetus (quartile 1, reference; quartile 2, RR 1.536 [95% CI 0.784,3.009]; quartile 3, 2.489 [1.336,4.637]; and quartile 4, 2.489 [1.291,4.798)]). The detailed results were showed in **Supplementary Table 2**.

**Table 3:** Association between FT4/TSH ratio quartiles and GDM.

Variables	Quartile 2	Quartile 3	Quartile 4
Total	374	372	371
Crude	1.070(0.746,1.536)	1.629(1.155,2.297)	1.364(0.961,1.937)
*P* value	0.712	0.005	0.083
Multivariable-adjusted^1^	1.171(0.806,1.703)	1.718(1.204,2.453)	1.461(1.010,2.113)
*P^1^* value	0.407	0.003	0.044
Multivariable-adjusted^2^	1.219(0.824,1.803)	1.873(1.276,2.748)	1.608(1.073,2.409)
*P^2^* value	0.322	0.001	0.021
Female	146	146	154
Crude	0.868(0.492,1.530)	1.058(0.609,1.838)	1.138(0.662,1.954)
*P* value	0.624	0.842	0.640
Multivariable-adjusted^1^	0.908(0.505,1.632)	1.138(0.643,2.013)	1.167(0.655,2.079)
*P^1^* value	0.748	0.657	0.601
Multivariable-adjusted^2^	0.826(0.447,1.524)	1.143(0.616,2.120)	1.200(0.639,2.525)
*P^2^* value	0.540	0.672	0.571
Male	154	166	151
Crude	1.184(0.689,2.036)	1.891(1.143,3.128)	1.413(0.831,2.405)
*P* value	0.541	0.013	0.202
Multivariable-adjusted^1^	1.212(0.694,2.118)	1.917(1.143,3.216)	1.509(0.868,2.622)
*P^1^* value	0.499	0.014	0.144
Multivariable-adjusted^2^	1.304(0.719,2.366)	2.154(1.221,3.797)	1.645(0.888,3.049)
*P^2^* value	0.383	0.008	0.114

Data are RR(95% CI).

^1^Adjusted for age and prepregnant body mass index.

^2^Adjusted for age, prepregnant body mass index, lipids and family history.

Since the rate of the-follow-up reached up to 16.8% in the present study, sensitivity analysis was used to evaluated the effect of the follow-up (**Supplementary Table 3**). We supposed all the rest of pregnant women successfully delivered. No matter all the rest of infants were male or female, the results were consistent with the primary conclusions.

## Discussion

In this prospective bi-center cohort study, we observed FT4/TSH ratio was closely associated with glucose and lipids metabolisms as well as insulin resistance during the first trimester, whereas only the third quartile of FT4/TSH ratio in women carrying male fetus was an independent risk factor of GDM. To our knowledge, this is the first study to examine the association between FT4/TSH ratio in the first trimester and the incidence of GDM as well as fetal sex specific difference in women with euthyroid function.

Gestational diabetes mellitus is one of the most common chronic pregnancy diseases affecting the health of millions of women worldwide (10, 11) and the prevalence of GDM is increasing. GDM not only causes adverse pregnancy outcomes such as preeclampsia and macrosomia but also increases the risk of developing type 2 diabetes later in life (12, 13). The international diabetes federation (IDF) reported the pooled global standardized prevalence of GDM was 14.0% and the regional standardized prevalence of GDM varied from 7.1% to 27.6% (14). The incidence of GDM in this cohort was up to 23%, which was relatively higher than average level in China. One possible cause might be the good economic level and living conditions in Beijing. Well-documented risk factors for GDM include advanced maternal age, lipids level, family history of diabetes and being overweight or obese (15, 16). Maternal age, BMI, the percentage of family history of diabetes glucose and lipids levels as well as insulin resistance were higher in GDM group, which was similar to the previous studies.

It is well acknowledged that hypothyroidism and hyperthyroidism can both affect glucose and lipids metabolism (17). Previous studies indicated thyroid dysfunction and diabetes mellitus are closely linked since thyroid hormones modulated by hypothalamic-pituitary-thyroid axis has an impact on glucose homeostasis (18) and insulin sensitivity can influence the feedback of thyroid hormones in turn (19). Prevalence of thyroid disorders in patients with diabetes mellitus is relatively high and it seems the incidence of diabetes mellitus is also higher in patients with thyroid dysfunction (20). However, some studies found euthyroid function might also be associated with risk of diabetes mellitus (21) and the cross-sectional association between thyroid hormones sensitivity and diabetes or prediabetes mellitus has been pointed out (22, 23). Even subtle changes in the levels of serum TSH and THs within the physiological range can induce insulin resistance or diabetes (24, 25), whereas the mechanism of this is still unclear. The relationship between euthyroid function and lipids metabolism has also been reported. Wang et al. found increased TSH levels and FT3/FT4 ratio were significantly associated with higher TC and LDL level (26). The authors performed a 2-sample bidirectional Mendelian randomization using summary statistics from large-scale genome-wide association studies of thyroid function and found higher TSH or lower FT4 within reference range were associated with increased TC and LDL-C (27).

Production of the thyroid hormones increases by nearly 50% during pregnancy and the daily iodine requirement also increases 50% to balance thyroid function. The burden of thyroid dysfunction can occur in many pregnant women and has a profound impact (28). Maternal thyroid function in pregnancy is closely associated with gestational complication and offspring outcomes. Recent birth-cohort studies suggest that even mild degrees of thyroid dysfunction may be linked with a range of late cognitive and behavioral effects in childhood and adolescence (29). Indeed, thyroid function might influence metabolism status even within normal range whereas the relationship between thyroid autoantibodies and GDM was controversial. There was an inverse dose-response association of maternal TSH and FT4 within the normal range with birthweight—higher FT4 concentrations are associated with lower birth weight, even within the normal range; but for TSH concentrations, the associations with birth weight were less evident and not present within the normal range (30). Previous studies reported TPOAb positivity was related to many adverse outcomes, such as miscarriage, premature delivery and low birth weight (31), but the relationship between TPOAb and GDM are not consistent. Yang et al. reported the prevalence of TPOAb positivity among 5,2027 pregnant women was 10%, a little bit lower than the 12.4% reported in an American study (31). In this cohort study, the prevalence of positive TPOAb was 11.3%, which was similar to previous studies. Sitoris et al. found the incidence of GDM was 26.1% in women with positive TPOAb, which was much higher than that in women with negative TPOAb(18.9%) (32). Higher TSH or lower FT4 levels were associated with an increased risk of GDM in assisted pregnancies for patients with positive TPOAb (33). Whereas Montaner et al. reported they have not identified TPOAb positivity in early pregnancy as a predictor of GDM (34). The percentage of positive TPOAb was no significant difference between GDM and NGT group in this cohort study, which was consistent with the latter one. An retrospective study included 40,156 pregnant women demonstrated an L-shaped association between maternal FT4 levels and GDM (35), while another study showed that higher FT3 levels and FT3/FT4 ratios were associated with increased GDM risk (36). We found although TSH and FT4 concentration were no difference between GDM and NGT group, FT4/TSH ratio was significantly higher in GDM group, which has not been noticed in previous studies. We speculated the differences of TSH and FT4 among pregnant women with euthyroid function in GDM group and NGT group were extremely slight, but FT4/TSH ratio can amplify the subtle difference, leading to the positive results.

Further analysis found FT4/TSH ratio was an independent risk factor of GDM but it was sex-specific of fetus. The mechanism was unclear and one of the possible pathology is the personalized thyroid hormones sensitivity among pregnant women and different hCG levels between women carrying male or female fetus (37, 38). Thyroid hormones and thyrotropin are inversely correlated under the negative feedback loop of hypothalamic-pituitary-thyroid axis, while normal thyroid hormones metabolism and action require adequate cellular receptors. The high thyroid hormones combined with high TSH represents an acquired resistance to thyroid hormones in the general population and thyroid hormones sensitivity is supposed to influence the metabolic status even in euthyroid population. Human chorionic gonadotropin (hCG)—a placental glycoprotein hormone—is lower in maternal circulation in the case of a male than a female fetus, which can stimulate the TSH receptor, increasing thyroid hormone production and resulting in a subsequent reduction in serum TSH concentration, especially in the first trimester (39–41). Some researchers observed that lower serum human chorionic gonadotrophin (hCG) levels during the first trimester were associated with a higher prevalence of GDM, with FT4 as a mediator (37). Vrijkotte et al. found sexual dimorphism appears to be present in the relationship between maternal thyroid metabolism and fetal intrauterine growth, with stronger associations in male infants (7). Besides, maternal GDM also influence thyroid hormone receptor of the human placenta in a sex- and cell-type specific manner (42). It is possible that the association of FT4/TSH ratio with the risk of GDM in women carrying male fetus is partly related to the above multiple factors complex interaction. Based on the above research, we can put forward a reasonable hypothesis. Both hCG and thyroid hormone are associated with the risk of GDM——relatively higher hCG might decrease the risk of GDM through multiple mechanisms such as immunomodulatory effect, anti-oxidative stress, anti-inflammatory and so on; thyroid function might have bidirectional regulation on glucose metabolism (37, 43, 44). High hCG during pregnancy can also affect the thyroid function at the same time, thus fluctuating FT4 and TSH might increase or decrease the risk of GDM according to the degree of volatility. During the pregnancy, hCG in women carrying female fetus is higher than those carrying male fetus, which means the weight coefficient of hCG for GDM in women carrying female fetus is higher and the effect of FT4/TSH ratio is weakened, leading to the sex-specific effect. However, further studies are needed since the hCG was not detected in this study and the sex-specific effect of FT4/TSH ratio and GDM has rarely been considered in previous studies.

Pregnancy-related hormone can affect the synthesis and metabolism of lipids, resulting in the physiological elevation of serum lipids, which can increase the risk of GDM, pregnant hypertension, preterm birth, LGA and even congenital cardiac disease of infants (45). Therefore, the management of blood lipids during pregnancy can also not be ignored. But the results about the relationship between thyroid function and lipid levels during early pregnancy is not consistent yet. Mehran et al. pointed out that FT4 was closely related to metabolic indicators, such as TC and TG levels, and lower FT4 levels may increase the risk of developing metabolic syndrome (46). Knight noted that FT4 level was distinctly negatively associated with BMI and TG but not with TC; however, TSH level was not correlated with any of these metabolic parameters (47). In our study, the relationship between FT4/TSH ratio and TC, TG, HDL-C as well as LDL-C was inverted U-shape, which was different from the previous study. On the one hand, lipids levels were unstable, which were closely associated with diet and other confounding factors; on the other hand, FT4/TSH ratio might have bidirectional influence on lipids levels, suggesting the complex lipid profile alterations during pregnancy and further studies are needed.

The innovation of this study was lied in the following aspects. First, this was the first time that fetus sex as an intermediary factor was shown to mediate the relationship between thyroid function and GDM based on a large sample. Second, this study was a prospective bi-center study with little information bias, and the results obtained were more reliable than those of a retrospective study. Third, we excluded patients with pregestational diabetes and thyroid disease to reduce the interference. However, our study also had limitations. First, the lost rate of follow-up in this study was higher than expected, which mainly because of pandemic of covid-19 impeding the regular follow-up of participants in designated hospitals. Although the sensitivity analysis was conducted, the loss of follow-up bias was difficult to be avoid completely. Second, although we adjusted for multiple covariates and PSM were used to control confounding factors, there still might be confounding bias such as environmental exposures and lifestyle factors that contribute to the observed associations (48–50). Third, there was no opportunity to investigate associations with free triiodothyronine since it was not measured in more than half pregnant women in this study. Therefore, we cannot investigate whether there were any T3–T4 conversion effects. Serum hCG level was also not recorded in the present study, thus the biological mechanisms behind this sex-specific effect can only be speculated according to previous studies. In addition, this was a bi-center prospective study, and its results may not be applicable to women in other regions. Therefore, future studies should be conducted on a broader population basis to increase the reliability and universality of the results.

## Conclusions

In conclusions, this study found that thyroid function even in normal range is closely related to glucose and lipids metabolisms during the first trimester. FT4/TSH ratio in the first trimester is an independent risk factor of GDM with sexual dimorphism, but it needs further study.

## Data Availability

The datasets presented in this article are not readily available because the datasets used and/or analyzed during the current study are available from the first author or corresponding author on reasonable request. Requests to access the datasets should be directed to Shuoning Song, shuoningsong@qq.com.
