# Supplementary material for: Thyroid FT4-to-TSH ratio in the first trimester is associated with gestational diabetes mellitus in women carrying male fetus: a prospective bi-center cohort study
Source: Front Endocrinol (Lausanne). 2024 Nov 29;15:1427925. doi: 10.3389/fendo.2024.1427925 (PMC11637856; doi:10.3389/fendo.2024.1427925)
Supplement: Supplementary file 1 [file DataSheet1.docx]

**Supplemental Tables**

**Supplemental Table 1. Baseline characteristics, neonatal characteristics and perinatal outcomes according to women carrying female or male fetus**

| Variables | Female | Male | *P* value |
| --- | --- | --- | --- |
| Baseline characteristics | n=582 | n=655 |  |
| Age(year) | 30.83±3.97 | 30.57±4.05 | 0.264 |
| preBMI(kg/m2) | 22.16±3.20 | 21.95±2.97 | 0.238 |
| BMI in 2nd trimester | 24.48±3.19 | 24.56±3.04 | 0.625 |
| BMI at delivery | 26.80±3.22 | 26.93±3.03 | 0.484 |
| Family history of diabetes mellitus | 14.4%(84) | 14.7%(96) | 0.911 |
| Previous adverse pregnancy outcomes | 19.9%(116) | 19.1%(125) | 0.707 |
| SBP(mmHg) | 117.12±8.62 | 116.59±8.68 | 0.289 |
| DBP(mmHg) | 74.31±6.73 | 74.57±6.58 | 0.503 |
| ALT(U/L) | 13.06(10.00,19.67) | 13.00(10.00,18.00) | 0.412 |
| AST(U/L) | 16.10(14.18,19.00) | 16.00(14.00,19.00) | 0.393 |
| TBil(umol/L) | 10.70(8.90,13.20) | 10.70(8.60,13.30) | 0.647 |
| TC(mmol/L) | 3.89(3.44,4.44) | 3.94(3.47,4.53) | 0.152 |
| TG(mmol/L) | 0.83(0.63,1.17) | 0.84(0.65,1.15) | 0.689 |
| HDL-C(mmol/L) | 1.44(1.21,1.63) | 1.43(1.26,1.66) | 0.212 |
| LDL-C(mmol/L) | 1.97(1.67,2.39) | 2.05(1.70,2.45) | 0.059 |
| Glucose(mmo/L) | 4.50(4.10,4.90) | 4.50(4.20,5.00) | 0.490 |
| Insulin(uU/mL) | 6.60(4.60,9.30) | 6.40(4.40,9.10) | 0.316 |
| C-Peptide(ng/dL) | 0.83(0.62,1.14) | 0.85(0.65,1.13) | 0.450 |
| HOMA-IR | 1.30(0.88,1.92) | 1.30(0.86,1.85) | 0.416 |
| OGTT |  |  |  |
| FBG(mmol/L) | 4.66(4.44,4.90) | 4.69(4.42,4.92) | 0.810 |
| 1-h BG(mmol/L) | 7.67(6.61,8.77) | 7.67(6.55,8.69) | 0.575 |
| 2-h BG(mmol/L) | 6.45(5.77,7.38) | 6.65(5.81,7.38) | 0.192 |
| GDM | 23.2%(135) | 22.9%(150) | 0.902 |
| FT4(ng/dL) | 1.25(1.13,1.40) | 1.23(1.10,1.38) | 0.065 |
| TSH(uIU/mL) | 1.60(0.91,2.42) | 1.69(1.09,2.48) | 0.051 |
| FT4/TSH ratio | 5.84(0.73,13.31) | 5.12(0.63,11.52) | 0.023 |
| TPOAb(IU/mL) | 13.7%(72/525) | 10.7%(63/591) | 0.118 |
| **Perinatal outcomes** | n=582 | n=655 |  |
| PIH | 7%(47) | 8.5%(56) | 0.763 |
| Premature delivery | 4.1%(24) | 3.5%(23) | 0.574 |
| Caesarean delivery | 27.3%(159) | 37.3%(244) | <0.001 |
| PPH | 12.7%(74) | 15%(98) | 0.254 |
| **Neonatal outcomes** | n=582 | n=655 |  |
| Birth age(weeks) | 39.43(38.57,40.14) | 39.29(38.57,40.00) | 0.208 |
| Birth weight (g) | 3225(2930,3460) | 3370(3100,3650) | <0.01 |
| LGA | 10.8%(63) | 14%(92) | 0.088 |
| SGA | 8.1%(47) | 7.5%(49) | 0.696 |

**Supplemental Table 2. Association between FT4/TSH ratio quartiles and GDM after PSM**

| Variables | Total | GDM | NGT | *P* value |
| --- | --- | --- | --- | --- |
| n | n=650 | n=229 | n=421 |  |
| Age(year) | 31.12±3.91 | 31.32±3.74 | 31.00±4.00 | 0.336 |
| preBMI(kg/m2) | 22.24±2.88 | 22.41±3.07 | 22.15±2.77 | 0.281 |
| Family history of diabetes mellitus | 15.4%(100) | 15.3%(35) | 15.4% (65) | 0.958 |
| TC(mmol/L) | 3.93(3.44,4.50) | 3.94(3.40,4.50) | 3.93(3.47,4.47) | 0.975 |
| TG(mmol/L) | 0.84(0.65,1.19) | 0.86(0.69,1.20) | 0.84(0.63,1.16) | 0.104 |
| HDL-C(mmol/L) | 1.42(1.22,1.63) | 1.43(1.21,1.59) | 1.42(1.23,1.65) | 0.593 |
| LDL-C(mmol/L) | 2.04(1.70,2.46) | 2.03(1.68,2.52) | 2.04(1.75,2.43) | 0.717 |

| Variables | Quartile 2 | Quartile 3 | Quartile 4 |
| --- | --- | --- | --- |
| Total | 149 | 172 | 165 |
| Crude | 1.070(0.746,1.536) | 1.629(1.155,2.297) | 1.364(0.961,1.937) |
| *P* value | 0.840 | 0.052 | 0.083 |
| Female | 70 | 74 | 88 |
| Crude | 0.671 (0.334,1.347) | 0.891(0.455,1.745) | 0.720(0.374,1.385) |
| *P* value | 0.262 | 0.737 | 0.325 |
| Male | 79 | 98 | 77 |
| Crude | 1.536(0.784,3.009) | 2.489(1.336,4.637) | 2.489(1.291,4.798) |
| *P* value | 0.211 | 0.004 | 0.006 |

Data are RR(95% CI).

**Supplemental Table 3. Association between FT4/TSH ratio quartiles and GDM after sensitivity analysis**

| Variables | Quartile 2 | Quartile 3 | Quartile 4 |
| --- | --- | --- | --- |
| Female | 206 | 204 | 215 |
| Crude | 0.980(0.603,1.593) | 1.428(0.891,2.288) | 1.307(0.818,2.089) |
| *P* value | 0.936 | 0.138 | 0.263 |
| Multivariable-adjusted[^1^](https://care.diabetesjournals.org/content/37/7/1878.long#fn-9) | 1.128(0.679,1.871) | 1.557(0.953,2.545) | 1.407(0.853,2.320) |
| *P*[*^1^*](https://care.diabetesjournals.org/content/37/7/1878.long#fn-9) value | 0.642 | 0.077 | 0.181 |
| Multivariable-adjusted[^2^](https://care.diabetesjournals.org/content/37/7/1878.long#fn-9) | 1.114(0.658,1.885) | 1.667(0.984,2.823) | 1.556(0.903,2.681) |
| *P*[*^2^*](https://care.diabetesjournals.org/content/37/7/1878.long#fn-9) value | 0.688 | 0.057 | 0.111 |
| Male | 213 | 223 | 214 |
| Crude | 1.226(0.767,1.960) | 2.132(1.369,3.319) | 1.528(0.963,2.425) |
| *P* value | 0.395 | <0.001 | 0.072 |
| Multivariable-adjusted[^1^](https://care.diabetesjournals.org/content/37/7/1878.long#fn-9) | 1.171(0.806,1.703) | 1.718(1.204,2.453) | 1.461(1.010,2.113) |
| *P*[*^1^*](https://care.diabetesjournals.org/content/37/7/1878.long#fn-9) value | 0.407 | 0.003 | 0.044 |
| Multivariable-adjusted[^2^](https://care.diabetesjournals.org/content/37/7/1878.long#fn-9) | 1.505(0.895,2.532) | 2.523(1.531,4.157) | 1.911(1.120,3.260) |
| *P*[*^2^*](https://care.diabetesjournals.org/content/37/7/1878.long#fn-9) value | 0.123 | <0.001 | 0.018 |

Data are RR(95% CI).

^1^Adjusted for age and prepregnant body mass index.

^2^Adjusted for age, prepregnant body mass index, lipids and family history.
